# Policy‐induced selection bias in pharmacoepidemiology: The example of coverage for Alzheimer's medications in British Columbia

**DOI:** 10.1002/pds.4804

**Published:** 2019-07-03

**Authors:** Anat Fisher, Greg Carney, Ken Bassett, K. Malcolm Maclure, Colin R. Dormuth

**Affiliations:** ^1^ Department of Anesthesiology, Pharmacology and Therapeutics University of British Columbia Vancouver British Columbia Canada; ^2^ Department of Family Practice University of British Columbia Vancouver British Columbia Canada

**Keywords:** Alzheimer's disease, cholinesterase inhibitors, drug reimbursement, interrupted time series analysis, pharmacoepidemiology, reimbursement policy, selection bias

## Abstract

**Purposes:**

To assess the impact of a government‐sponsored reimbursement policy for cholinesterase inhibitors (ChEIs) on trends in physician visits with a diagnosis of Alzheimer's disease (AD).

**Methods:**

Longitudinal population‐based study using interrupted time series methods. British Columbia outpatient claims data for individuals aged 65 and older were used to compute monthly AD visit rates and examine the impact of the ChEI reimbursement policy on the coding of AD. We examined trends in the number of patients with AD visits, the number of AD visits per patient, and visits with “competing” diagnoses (mental, neurological, and cerebrovascular disorders and accidental falls). Finally, we described demographic and clinical features of diagnosed patients.

**Results:**

We analyzed 1.9 million AD visits. Faster growth in recorded AD visits was observed after the policy was implemented, from monthly growth of 7.5 visits per 100 000 person‐months before the policy (95% confidence interval [CI], 6.1‐8.9) to monthly growth of 16.5 per 100 000 person‐months after the policy (95% CI, 14.8‐18.3). After the implementation of the policy, we observed increased growth in the number of patients with recorded AD visits and the number of AD visits per patient, as well as a shift in diagnoses away from mental diseases and accidental falls to AD (diagnosis substitution).

**Conclusions:**

British Columbia's reimbursement policy for ChEIs was associated with a significant acceleration in Alzheimer's visits. Evaluations of health services utilization and clinical outcomes following drug policy changes need to consider policy‐induced influences on the reliability of the data used in the analysis.

**KEY POINTS**

- This study assessed the effect of a new government‐sponsored reimbursement policy for Alzheimer's medications in British Columbia on the rate of physician visits with a diagnosis of Alzheimer's disease.
- The new policy was associated with an increase in growth of observed Alzheimer's visits, from a monthly growth of 7.5 visits per 100 000 person‐months before the policy to 16.5 per 100 000 person‐months after the policy.
- This policy was also associated with faster growth in the number of patients with an Alzheimer's disease diagnosis, faster growth in the number of Alzheimer's visits per patient, and substitution of diagnosis codes from mental disorders and accidental falls to Alzheimer's disease.
- The policy implementation was associated with changes in the characteristics of diagnosed patients and could bias assessment of health services utilization and clinical outcomes associated with this policy.

## 1 INTRODUCTION

Administrative health claims data are generated after encounters with the health care system and are collected for administrative or billing purposes.^1^ These data have been used in a growing number of pharmacoepidemiology and health policy studies.^2^, ^3^ One element of claims data is diagnosis codes. Diagnostic information is typically recorded in claims for outpatient physician visits and is frequently used to define study populations and identify study outcomes. Inaccuracies in diagnostic coding have been discussed as an important source of bias^3^, ^4^; however, such inaccuracies were generally assumed to be constant over the study period, in spite of evidence that this might not be the case. Multiple situations have been shown to influence the utilization of specific diagnostic codes, including a change in codes or the coding system,^5^, ^6^ the introduction of new diagnostic criteria,^7^, ^8^ increased awareness by physicians and the public for a specific disease,^9^ and the substitution of codes for one disease with codes for another (diagnostic shift).^10^, ^11^, ^12^, ^13^, ^14^, ^15^, ^16^, ^17^

In the Canadian Province of British Columbia (BC), we identified a unique opportunity to examine the susceptibility of diagnostic coding behavior to a change in drug reimbursement policy. In October 2007, the provincial drug plan began covering the cholinesterase inhibitor medications (ChEIs: donepezil, galantamine, and rivastigmine) for patients with Alzheimer's disease (AD) as part of the Alzheimer's Drug Therapy Initiative (ADTI). The policy was implemented as part of an initiative for “coverage with evidence development,”^18^, ^19^, ^20^ and its details are presented elsewhere.^21^, ^22^ We sought to assess the effect of the ADTI reimbursement policy (aka “the policy”) on health services utilization and cost in AD patients. We undertook this analysis as a preliminary measure to understand the possible influence of the policy on diagnostic information captured in the database, prior to using the database to evaluate the policy.

## 2 METHODS

### 2.1 Study design and data source

We conducted a longitudinal population‐based study using interrupted time series analysis methods. We obtained administrative claims data from the BC Ministry of Health for the period 1 January 2001 to 31 December 2013. The anonymized data included records of fee‐for‐service payments to physicians and alternative providers, patient registration information and demographics, pharmacy records (PharmaNet), and hospital discharge records.

### 2.2 Physician visits for AD

Our study assessed the impact of the ADTI policy on diagnostic coding for AD in outpatient visits (“Alzheimer's visits”). Alzheimer's visits were defined as a physician fee‐for‐service visits with an International Classification Disease, version 9 (ICD‐9) code of 331, 290, 294, or 797 in any of the diagnostic fields. This definition was the only published AD definition validated on administrative data when this study was conducted,^23^ and its sensitivity and specificity were 86%.^24^, ^25^ Individuals under the age of 65 years were excluded because dementia is rare and often secondary to other diseases in that age group.^26^, ^27^ Crude and standardized Alzheimer's visit rates were computed on a monthly basis. Crude visit rates were calculated as the numbers of Alzheimer's visits per 100 000 person‐months of enrollment in the provincial medical plan. Directly standardized visit rates were computed to correct for variations in age and sex over time, using the 2007 British Columbia enrolled population as the reference population. Standardized visit rates were also corrected to a month‐length of 30 days.

### 2.3 Statistical methods

Interrupted time series analysis is considered the strongest, quasi‐experimental design to evaluate the longitudinal effects of an intervention, eg, health policy, particularly when the researcher does not manage the intervention.^28^, ^29^, ^30^ Applying this methodology, we included the following variables in our regression model: time in months, time after the ADTI policy, and a dichotomous variable for baseline level. Time in months, from January 2001 and onwards, ie, the trend before ChEI reimbursement policy, was included to correct for a secular trend in AD diagnostic coding. Time after the new policy from November 2007 onwards was included to test for the effect of the policy on top of any preexisting trend. The model assumed a linear trend. We added a dichotomous variable denoting “before” versus “after” the implementation of the policy. This dichotomous variable tests for a change in AD visits between the months immediately before and immediately after the policy, accounting for the prepolicy trend.^31^ In addition, we adjusted for autocorrelation and seasonality by including lag terms for up to 12 preceding months based on statistical significance (stepwise autoregression using the Yule‐Walker method, SAS BACKSTEP selection option). Finally, to allow for a delayed effect of the policy, we excluded data from the first three months after the policy was launched (November 2007‐January 2008). The analysis was conducted using SAS PROC AUROREG.^28^, ^29^, ^32^

### 2.4 Additional analyses

#### 2.4.1 ChEI reimbursement policy and characteristics of incident cases

We explored possible differences in the characteristics of incident Alzheimer's patients before and after the reimbursement policy implementation. Incident cases were defined based on the first of two outpatient physician claims within 18 months or first hospital discharge with Alzheimer's diagnosis between January 2001 and December 2012. We excluded patients based on an earlier Alzheimer's diagnosis, the absence of continuous enrollment within the preceding 18 months, or under 65 years old. We described the characteristics of incident cases identified each year and presented the proportions of patients with different categories for the following variables: age, gender, income, comorbidities (Romano score based on data during the year before diagnosis^33^), and the specialty of the diagnosing physician.

#### 2.4.2 ChEI reimbursement policy and additional visits/patients’ parameters

We further tested for the effect of the ChEI reimbursement policy on several monthly parameters for individuals aged 65 and older using interrupted time series analysis. Visit ratio was defined as the number of physician visits with an Alzheimer's diagnosis divided by 100 000 total physician visits. Visit density was defined as the number of physician visits with an Alzheimer's diagnosis per 100 individuals with such visits. Alzheimer's patients were the number of patients with a physician visit with an Alzheimer's diagnosis per 100 000 person‐months of enrollment. Lastly, Alzheimer's administrative incidence was defined as the first physician visit or hospital discharge with Alzheimer's diagnosis in patients with at least 18 months of continuous enrollment and no ChEI prescription during this period. Incidence was calculated per 100 000 person‐months of enrollment.

#### 2.4.3 ChEI reimbursement policy and diagnosis substitution

We considered that the ChEI reimbursement policy might have been associated with diagnosis substitution, ie, the substitution of codes for one diagnosis by codes for another disease. Specifically, for patients with the same clinical presentation, we considered whether physicians increased the use of AD coding after the policy over other codes that had been previously used. We tested for diagnosis substitution from four “competing” disease categories that may be relevant: mental disorders (ICD‐9 codes 290‐319), neurological disorders (ICD‐9 codes 320‐359 or 430‐438), cerebrovascular disorders (ICD‐9 codes 430‐438), and accidental falls (including orthopedic trauma, ICD‐9 codes E880‐E888, 800‐849). In the absence of accepted methodology to identify diagnosis substitution in epidemiology studies, we a priori defined diagnosis substitution as a combination of two criteria: the first is a decrease in the coding for the “competing” diseases, and the second is an increase in the ratio of Alzheimer's visits to the “competing” diseases. The analysis included a series of two interrupted time series analyses for each “competing” disease categories. For the first criterion, we analyzed visit rates with “competing” disease diagnoses and required significantly fewer visits with “competing” diseases after the new policy, ie, smaller slope or lower baseline level after policy initiation. For the second criterion, we analyzed the visit rate ratio: the product of rates of physician visits with an Alzheimer's diagnosis divided by the rates of visits with a diagnosis of a “competing” disease. For this criterion, we required a larger slope or higher baseline level after policy initiation.

## 3 RESULTS

### 3.1 ChEI reimbursement policy and Alzheimer's visits

**Figure 1:**
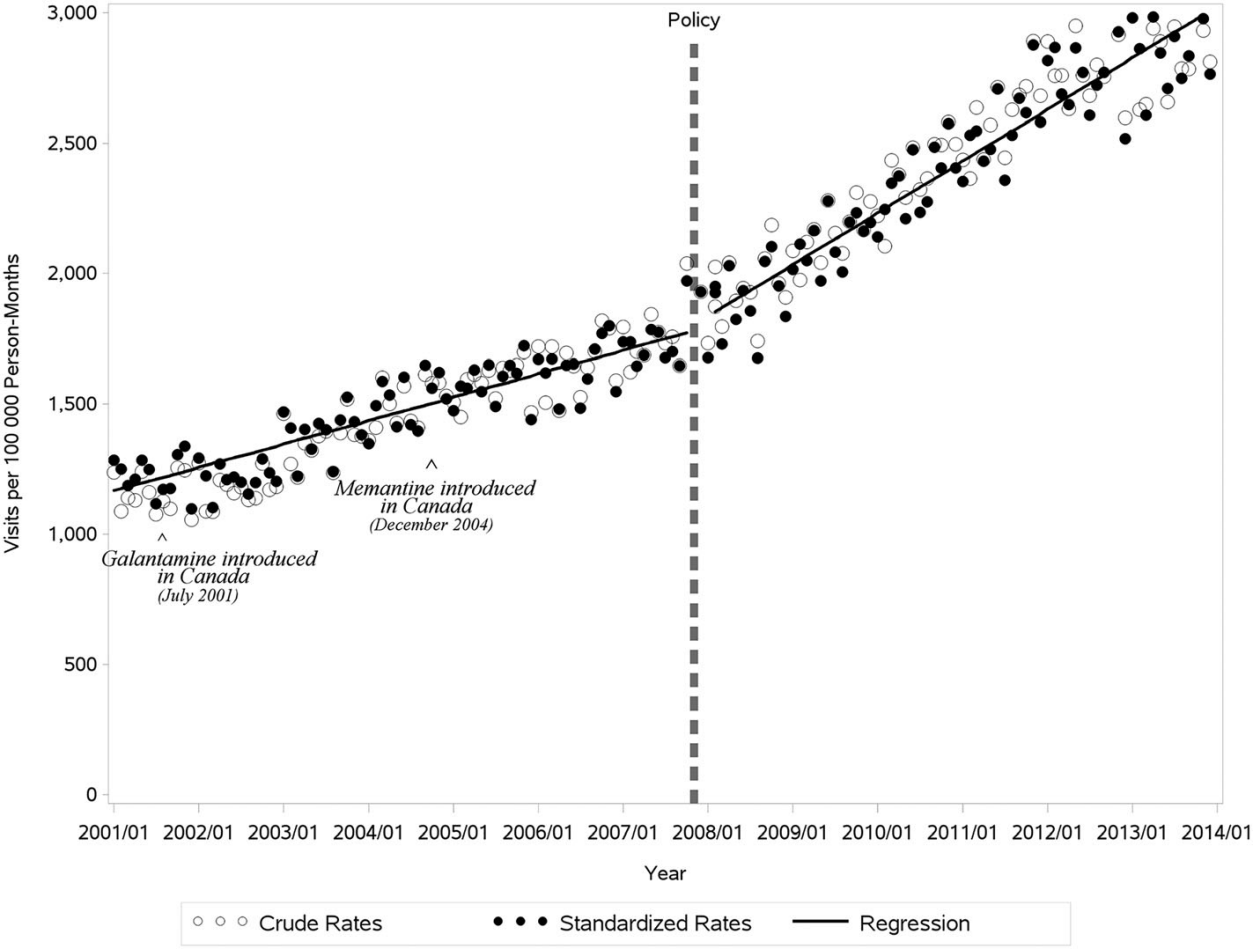
Alzheimer's visits per 100 000 patient‐months age 65 and older, British Columbia. Horizontal dashed line represents the initiation of cholinesterase inhibitor (ChEI) cost coverage

Between 2001 and 2013, 158.5 million physician visits were recorded in BC for patients aged 65 or older. About 1.9 million of these visits were coded with an AD diagnosis. The average rate of Alzheimer's visits increased from 6 160 visits per month in 2001 to 21 901 visits per month in 2013. During 13 years of follow‐up, we observed a 2.5‐fold increase in crude and standardized rates of Alzheimer's visits (Figure 1). The mean observed annual growth (±1 standard deviation) in the standardized rates of visits was 6.2% (5.0%) and 8.7% (2.7%) in the periods before and following the ChEI reimbursement policy.

In the interrupted times series analysis, we observed a significant association of the ChEI reimbursement policy with trends of physician visits with an Alzheimer's diagnosis (Figure 1, Table 1). Before the policy was implemented, the monthly growth in standardized Alzheimer's visits (the slope) was +7.5 visits per 100 000 person‐months (95% confidence interval [CI], 6.1‐8.9). After the policy was implemented, the slope increased by 9.0 (95% CI, 6.6‐11.5), to a monthly growth of +16.5 (95% CI, 14.8‐18.3) without a significant change in the baseline level of visits.

**Table 1:** Results of interrupted time series regression

Outcome	Baseline Level (Intercept)	Slope Before the New Policy	Slope After the New Policy	Level Change at Policy Implementation	Slope Difference
Alzheimer's Visits†	1166.0 (1096.5,1236.1)	1.5 (6.1,8.9)	16.5 (14.8,18.3)	23.3 (−58.3,104.9)	9.0 (6.6,11.5)*
Visit ratio	803.9 (771.4,836.5)	3.1 (2.5,3.8)	9.2 (8.3,10.0)	22.5 (−17.9,62.9)	6.1 (4.9,7.2)*
Visit density	148.1 (145.1,151.1)	0.2 (0.1,0.2)	0.3 (0.2,0.4)	−4.5 (−8.5,−0.4)*	0.1 (0.04,0.2)*
Alzheimer's patients†	792.6 (746.9,838.3)	4.0 (3.1,4.9)	7.3 (6.2,8.5)	45.8 (4.0,87.6)*	3.3 (1.6,5.0)*
Alzheimer's administrative incidence†	241.7 (231.8,251.4)	−0.8 (−0.9,−0.6)	0.1 (−0.1,0.2)	19.5 (10.9,28.2)*	0.7 (0.6,1.1)*

Alzheimer's visits, physician visits with Alzheimer's diagnosis codes; Visit ratio, the number of physician visits with an Alzheimer's diagnosis divided by 100 000 total physician visits; Visit density, the number of physician visits with an Alzheimer's diagnosis per 100 individuals with such visit; Alzheimer's patients, the number of patients with a physician visit with an Alzheimer's diagnosis code. Alzheimer's administrative incidence is based on the first physician visit or hospital discharge with an Alzheimer's diagnosis in patients with at least 18 months of continuous enrollment and no ChEI prescription during this period. Results are presented as estimated regression parameters (95% confidence interval).

† Per 100 000 patient‐months;

* Significant at the .05 probability level.

### 3.2 Additional analysis

**Figure 2:**
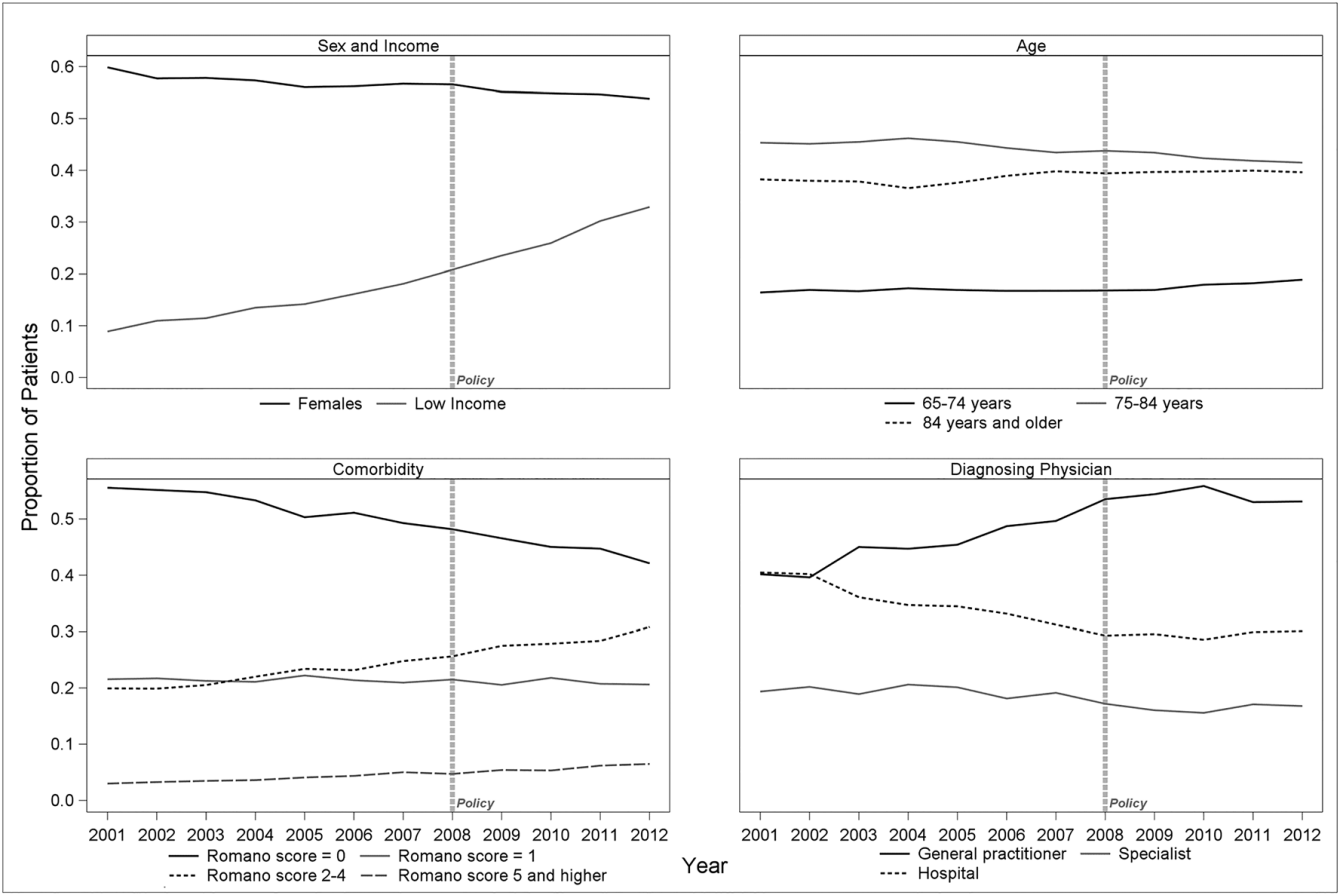
Cholinesterase inhibitor (ChEI) reimbursement policy and characteristics of incident cases. Data are presented for incidence cases of Alzheimer's disease and related dementias that were diagnosed between 2001 and 2012. In the absence of complete hospital data for the year 2013 at the time of analysis, the information for this year is not presented

We examined a possible association between the ChEI reimbursement policy and the demographic and clinical characteristics of incident cases. We observed an increase in the proportion of patients with multiple comorbidities and lower income after the ChEI reimbursement policy was launched (Figure 2). Both characteristics are important confounders in health service and health outcome research.^34^, ^35^, ^36^

**Figure 3:**
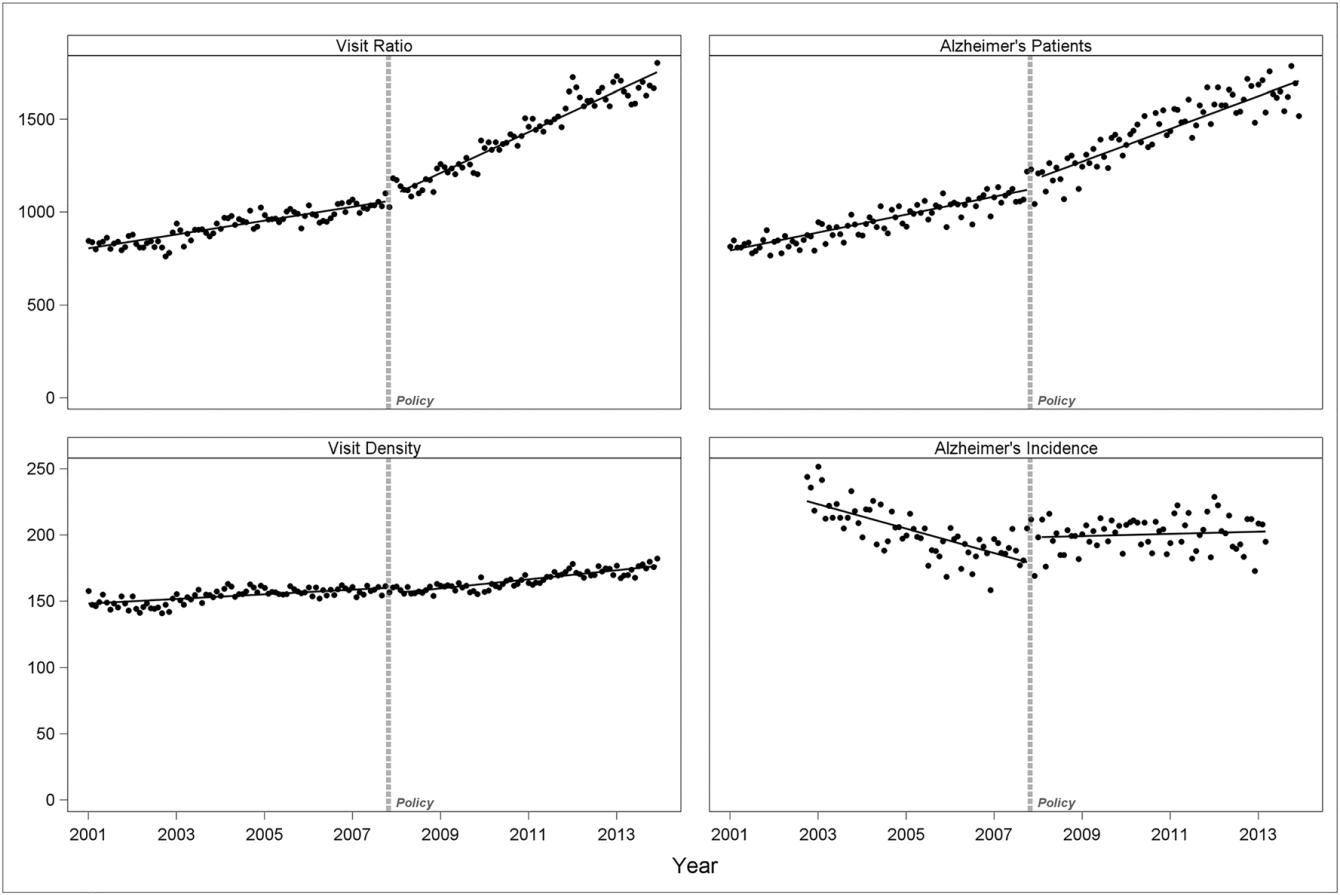
Cholinesterase inhibitor (ChEI) reimbursement policy and additional visits/patients' parameters. Visit ratio is the number of Alzheimer's visits per 100 000 all‐cause physician visits for individuals aged 65 or older in the province. Visit density is the number of Alzheimer visits per 100 individuals with such visits. Patients with Alzheimer's visits is defined per 100 000 person‐months of enrollment. Alzheimer's incidence is defined per 100 000 person‐months of enrollment, based on first physician visit or hospital discharge with Alzheimer's diagnosis in the data, in patients with at least 18 months of continuous enrollment and no cholinesterase inhibitor (ChEI) prescription during these 18 months

Next, we analyzed additional visits and patients' parameters. After the new reimbursement policy was implemented, we observed a significantly larger increase in three of the additional parameters studied: visit ratio (the number of physician visits with an Alzheimer's diagnosis divided by 100 000 total physician visits), visit density (the number of physician visits with an Alzheimer's diagnosis per 100 individuals with such visits), and Alzheimer's patients (the number of patients with a physician visit with an Alzheimer's diagnosis per 100 000 person‐months of enrollment) (Table 1 and Figure 3). We also compared Alzheimer's administrative incidence before and after the policy. We found that the new ChEI reimbursement policy was associated with a level increase of +19.5 (95% CI, 10.9‐28.2) incident cases per month. It was also associated with a change in direction of the slope, from a decreasing trend of −0.8 (95% CI, −0.9 to −0.6) incidence cases per 100 000 person‐months before the policy, to a constant trend of +0.1 (95% CI, −0.1 to 0.2) after the reimbursement policy was implemented (Table 1 and Figure 3).

**Figure 4:**
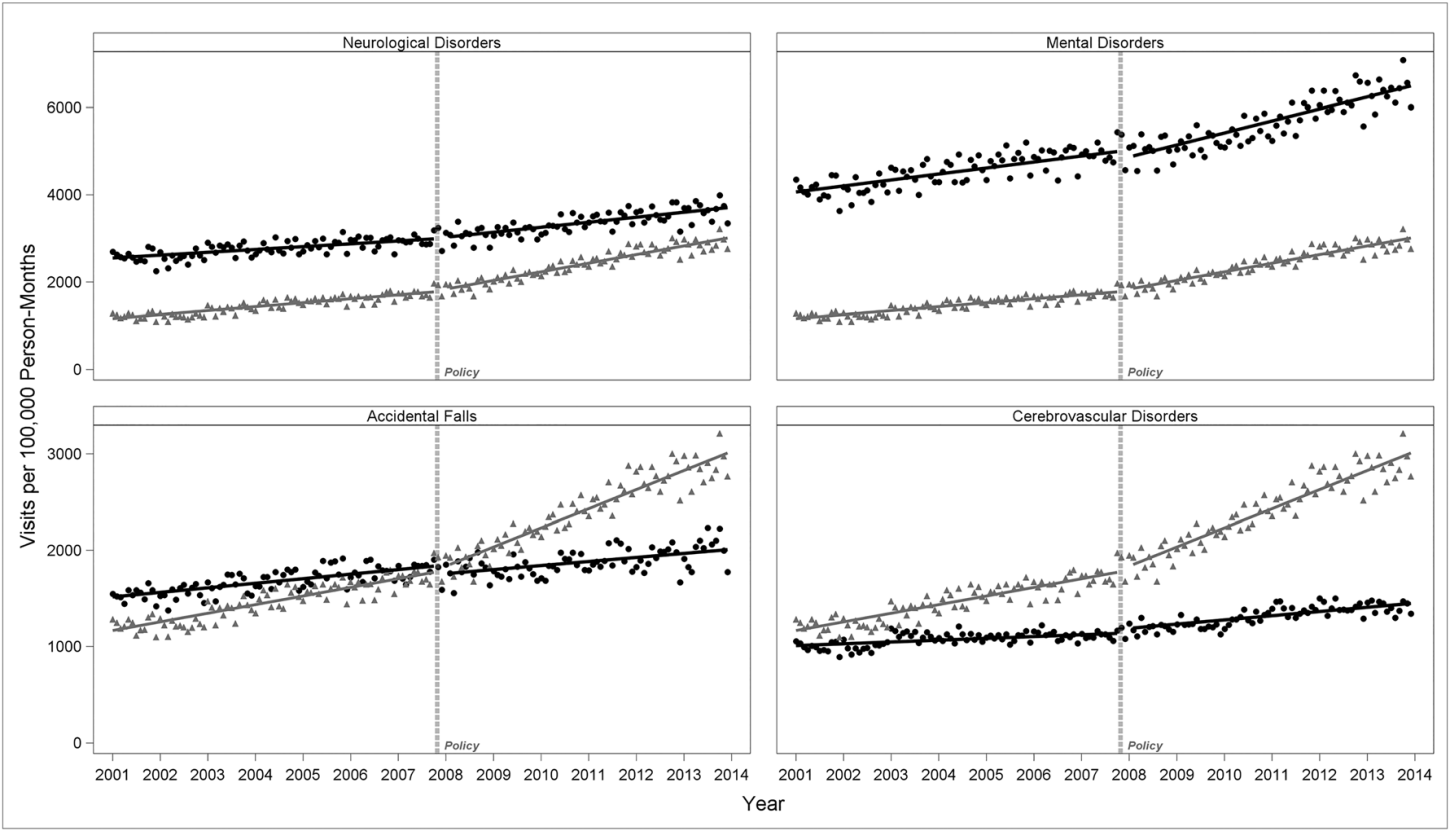
Cholinesterase inhibitor (ChEI) reimbursement policy and diagnosis substitution. Numbers are monthly visit rates, defined as the number of visits per 100 000 person‐months, in individuals age 65 and older. In Black ‐ observed monthly standardized rates (dots) and regression lines of visits with “competing” disease. In Gray ‐ observed monthly standardized rates (triangles) and regression lines of visits with Alzheimer's diseases

Finally, we checked whether physicians increased their use of Alzheimer's diagnosis coding over coding of other “competing” diseases after the new policy, ie, diagnosis substitution (Table 2 and Figure 4). On the basis of the two predefined criteria, we detected diagnosis substitution from mental disorders and accidental falls (Table 2). In both categories, the new policy was associated with a significant decrease in the baseline level of visits with the “competing” diseases and an increase in the baseline ratio of Alzheimer's visits to visits with “competing” diseases ratio.

**Table 2:** ChEI reimbursement policy and diagnosis substitution—results of interrupted time series regression

Outcome	Regression Outcome	Baseline Level (Intercept)	Slope Before the New Policy	Level Change at Policy Implementation	Slope Difference	Criterion	Criterion Applied?
Visits with diagnosis of metal disorders	Visits' rate†	4065.0 (3894.1,4235.4)	11.4 (8.1,14.8)	−181.6 (−360.4,−2.8)*	11.4 (5.5,17.3)	Fewer visits after policy initiation	Yes, level drop
	Rates ratio (x10^−3^) ‡	288.3 (275.5,301.2)	0.9 (0.6,1.1)	21.4 (7.6,35.2)*	0.4 (−0.1,0.8)	Increased rates ratio after policy initiation	Yes, level increase
Visits with diagnosis of neurologic disorders	Visits’ rate †	2551.0 (2459.7,2641.7)	5.4 (3.6,7.2)	13.2 (−92.2,118.7)	4.2 (1.0,7.3)	Fewer visits after policy initiation	No
	Rates ratio (x10^‐3^) ‡	458.7 (443.3,474.2)	1.7 (1.4,2.0)	12.1 (−9.2,33.4)	1.2 (0.7,1.7)*	Increased rates ratio after policy initiation	Yes, slope increase
Visits with diagnosis of cerebrovascular disorders	Visits’ rate †	1009.0 (960.2,1058.8)	1.6 (0.6,2.6)	44.2 (−20.2,108.6)	2.1 (0.4,3.8)	Fewer visits after policy initiation	No
	Rates ratio (x10^‐3^) ‡	1156.2 (1111.5,1200.9)	5.0 (4.1,5.9)	−0.4 (−67.1,66.3)	2.2 (0.6,3.7)*	Increased rates ratio after policy initiation	Yes, slope increase
Visits with diagnosis of accidental falls	Visits’ rate †	1515.0 (1483.0,1547.9)	4.0 (3.3,4.6)	−89.0 (−135.8,42.2)*	−0.5 (−1.6,0.7)	Fewer visits after policy initiation	Yes, level drop
	Rates ratio (x10^‐3^) ‡	767.0 (737.8,796.2)	2.5 (1.9,3.2)	67.4 (23.3,111.6)*	4.1 (3.1,5.1)*	Increased rates ratio after policy initiation	Yes, level increase and slope increase

Abbreviation: ChEI, cholinesterase inhibitor.

† Visits' rate defined as visits with “competing” diseases per 100 000 person‐months;

‡ Rates ratios are the products of the rate of Alzheimer's visits divided by the rate of visit with “competing” diseases;

* Significant at the .05 probability level.

## 4 DISCUSSION

In this study, we tested for the effect of a new drug reimbursement policy for Alzheimer's medications in British Columbia on the frequency in which the provincial physicians recorded Alzheimer's diagnosis during visits of individuals age 65 or older. Our findings reveal a temporal selection bias in patients with presumed AD who are eligible for study in epidemiologic analysis.

When studying the effect of a new intervention, such as a new health policy, we are often interested in estimating its effect on the relevant population, eg, individuals with a specific health or morbidity status. Our analysis of the British Columbia Alzheimer's medication policy naturally focused on patients with AD and related dementias and showed that a change in drug coverage had the potential to affect not only clinical and utilization outcomes in patients who are studied, as might be expected, but also the population of patients included in the study itself. This may cause bias when using a dynamic cohort in pharmacoepidemiological studies. A dynamic cohort in our example is a cohort to which patients continuously enter based on a first physician's visit or a hospital discharge with a diagnosis of AD. Dynamic cohort designs may be used to minimize biases caused by the duration of disease, to increase the sample size, or to deal with frequent dropping out from the study. In the current study, we showed that a drug policy can influence the number of patients entering the cohort, the reliability of the diagnosis (ie, the accuracy of the diagnosis code), and the distribution of covariates (ie, characteristics of patients entering the cohort). These effects of the policy could lead to selection bias when using a dynamic cohort design to study health care policy.

The true incidence of AD in the population, even if increasing, should have been reasonably stable and not prone to the sudden and dramatic changes that were shown in this analysis. The policy was associated with dramatically faster growth in the number of visits coded with AD even after accounting for secular trends, an increase that was composed of faster growth in the number of patients with Alzheimer's visits and visits per patient. These effects were independent of the number of all‐cause visits in the province. The faster growth in the number of patients with coded AD visits was also expressed as an increase in the estimated incidence of AD. Furthermore, we described differences in comorbidities and income between incident cases identified before the policy and those identified later; both are important sources of bias in health service research.^34^, ^35^, ^36^

Our outcomes were estimated based on administrative data, which include records that were created mainly for billing purposes. As a result, some inaccuracy is expected, and the use of specific diagnosis codes may be affected by different factors. Because we did not expect the new policy to cause a real change in the incidence trend, we suggest several possible explanations for the observed change in trends of Alzheimer's visits. First, the findings might have been caused by a time‐dependent change in the accuracy of Alzheimer's diagnostic coding (reliability). While we expected a decrease in the number of visits with a different diagnosis for some AD patients (false negatives), we could not rule out an increase in the number of AD diagnoses recorded for non‐AD patients (false positives). Unfortunately, we were unable to directly measure the AD coding accuracy in the BC data. Second, more patients were treated with ChEI medication when their costs were covered (data not shown). Patients on treatment were more likely to experience increased frequency of routine follow‐up visits,^37^ which were coded as AD. This explanation is supported by an increase in the frequency of visits per patient (visits density). Third, together with the reimbursement policy, the province initiated an educational program, the Dementia Education Strategy.^38^ Physicians may have been more alert to diagnosing AD and encouraging routine visits after participating in this professional development program. Last, the administrative requirements of applying for medication reimbursement or renewing it probably promoted an increase in the use of AD codes, which may have been associated with an increase in the frequency of visits per patient.

Our results are different from a previous BC study that examined the effect of drug coverage policy on visits.^39^ This published study estimated no effect of drug cost sharing on trends of visits with depression. The main difference between the studies is the direction of effect on copayment; in our study, it was lower after the policy, and in the previous study, it was higher. In addition, the previous study examined a more general policy that included a few medication groups. Trends in absolute numbers of visits with AD or dementia diagnosis codes have only been studied in a single study.^40^, ^41^ The researchers of that study estimated 18.2% annual growth in the number of physician visits with an AD diagnosis (ICD‐9 codes 290, 294, and 331) between 1998 and 2009 in patients aged 40 and older in the United States. The increase in the absolute number of visits was higher than what was observed in British Columbia. However, since the results of the American study did not include rates or proportions, it is difficult to estimate the extent of growth in AD caused by population growth or aging.

This study has several strengths. The Canadian health care system is based on the principles of fairness and equity, comprehensiveness, accessibility, and universality; hence, it is well suited to study the effects of new policies. We analyzed data from a population‐based databases in which data were collected prospectively in a systematic manner, and examined data had been collected over a long period of 13 years. Analyses of this sort are vulnerable to bias from cointerventions. In this specific instance, there may have been other programs and incentives that could have directly influenced trends of Alzheimer's visits during this period, such as education and guidelines for treating dementia patients^42^, ^43^ and incentives in treating patients with chronic diseases and complex clinical presentation.^44^, ^45^ While we were unable to validate the list of ICD codes used to identify Alzheimer's visits, we find the validation secondary to the main purpose of the study. Regardless of the accuracy of the AD codes, we demonstrated a change in trend related to the new drug reimbursement policy.

## 5 CONCLUSIONS

The observed increase in the number of physician visits with an AD diagnosis after the implementation of a government‐sponsored reimbursement policy for ChEI could present a challenge when studying other aspects of the new drug coverage policy. Policy‐induced influences on the selection of a study population could bias assessment of health services utilization and clinical outcomes in before‐after designs even when they include historical or concurrent control groups. We encourage researchers to critically evaluate the accuracy of diagnostic coding and trends and consider describing the effect of the policy on the cohort studied as part of the policy effect.

## ETHICS STATEMENT

The study protocol was approved by the Clinical Research Ethics Board of the University of British Columbia (H09‐01696) and the Human Research Ethics Board of the University of Victoria (08‐164).

## CONFLICT OF INTEREST

The authors declare no conflict of interest.
